# Severe South American Ocular Toxoplasmosis Is Associated with Decreased Ifn-γ/Il-17a and Increased Il-6/Il-13 Intraocular Levels

**DOI:** 10.1371/journal.pntd.0002541

**Published:** 2013-11-21

**Authors:** Alejandra de-la-Torre, Arnaud Sauer, Alexander W. Pfaff, Tristan Bourcier, Julie Brunet, Claude Speeg-Schatz, Laurent Ballonzoli, Odile Villard, Daniel Ajzenberg, Natarajan Sundar, Michael E. Grigg, Jorge E. Gomez-Marin, Ermanno Candolfi

**Affiliations:** 1 GEPAMOL, Centro de Investigaciones Biomédicas, Universidad del Quindío, Armenia, Colombia; 2 Institut de Parasitologie et Pathologie Tropicale, Fédération de Médecine Translationelle, Université de Strasbourg, Strasbourg, France; 3 Universidad del Rosario, Escuela de Medicina y Ciencias de la Salud, Departamento de Inmunología, Bogotá, Colombia; 4 Service d'Ophtalmologie, Hôpitaux Universitaires de Strasbourg, Strasbourg, France; 5 Centre National de Référence (CNR) Toxoplasmose/Toxoplasma Biological Resource Center (BRC), Centre Hospitalier-Universitaire Dupuytren, Limoges, France and INSERM UMR 1094, Neuroépidémiologie Tropicale, Laboratoire de Parasitologie-Mycologie, Faculté de Médecine, Université de Limoges, Limoges, France; 6 Laboratory of Parasitic Diseases, National Institutes of Health, National Institute of Allergy and Infectious Diseases (NIAID), Bethesda, Maryland, United States of America; McGill University, Canada

## Abstract

In a cross sectional study, 19 French and 23 Colombian cases of confirmed active ocular toxoplasmosis (OT) were evaluated. The objective was to compare clinical, parasitological and immunological responses and relate them to the infecting strains. A complete ocular examination was performed in each patient. The infecting strain was characterized by genotyping when intraocular *Toxoplasma* DNA was detectable, as well as by peptide-specific serotyping for each patient. To characterize the immune response, we assessed *Toxoplasma* protein recognition patterns by intraocular antibodies and the intraocular profile of cytokines, chemokines and growth factors. Significant differences were found for size of active lesions, unilateral macular involvement, unilateral visual impairment, vitreous inflammation, synechiae, and vasculitis, with higher values observed throughout for Colombian patients. Multilocus PCR-DNA sequence genotyping was only successful in three Colombian patients revealing one type I and two atypical strains. The Colombian OT patients possessed heterogeneous atypical serotypes whereas the French were uniformly reactive to type II strain peptides. The protein patterns recognized by intraocular antibodies and the cytokine patterns were strikingly different between the two populations. Intraocular IFN-γ and IL-17 expression was lower, while higher levels of IL-13 and IL-6 were detected in aqueous humor of Colombian patients. Our results are consistent with the hypothesis that South American strains may cause more severe OT due to an inhibition of the protective effect of IFN-γ.

## Author Summary

Ocular toxoplasmosis (OT), due to protozoan parasite *Toxoplasma gondii*, is a potential complication of both acquired and congenital infection, leading to visual impairment in numerous countries and being responsible for 30 to 50% of uveitis cases in immunocompetent individuals. In this study we confirmed the presence of more severe ocular toxoplasmosis in a tropical setting of Colombia, when compared to France. The main hypothesis for these clinical differences is based on the idea that severe disease in humans may result from poor host adaptation to neotropical zoonotic strains of *T. gondii* Indeed, our results are consistent with the hypothesis that South American strains may cause more severe OT due to an inhibition of the intraocular protective immune response.

## Introduction

Infection with the protozoan parasite *Toxoplasma gondii* is a leading cause of visual impairment in numerous countries, being responsible for 30 to 50% of uveitis cases in immunocompetent individuals [1]. Ocular toxoplasmosis (OT) is a potential complication of both acquired and congenital toxoplasmosis [2]. The incidence of ocular toxoplasmosis has been estimated in Colombia (Quindio region) to be of three new episodes by 100 000 inhabitants by year [3], while in British-born patients it has been estimated to be 0.4 cases per 100,000 population per year and the lifetime risk of disease to be 18 cases per 100,000 population [4].

In a Colombian study, 5.5% of the population in the province of Quindío exhibited retinochoroidal scars resulting from a postnatally acquired infection, with 20% of this group presenting reduced visual capacity. [3], [5]. In a retrospective study on uveitis conducted in 693 Colombian patients, 417 of whom had a definitive diagnosis, toxoplasmosis was the most frequent cause with 276 cases (39.8%) followed by idiopathic uveitis and toxocariasis [6].

Some differences between South American and European clinical case series were observed in terms of congenital transmission rates, probability of symptoms in congenital OT [7], [8], severity of ocular inflammation [9] and intraocular specific antibody levels [10]. However, no comparative clinical and biological studies have been performed yet in patients from both continents with laboratory-confirmed OT.

The population structure of *T. gondii* in North America and Europe includes three highly prevalent clonal lineages, Types I (haplogroup 1, Clade A), II (Haplogroup 2, Clade D), and III (haplogroup 3, Clade, C) which differ greatly in virulence in the mouse model. The vast majority of human and animal infections are caused by the relatively avirulent Type II strains. In contrast, heterogeneous atypical genotypes of *T. gondii* are associated with severe infections in humans in South America. They belong to various haplogroups: 4, 5, 8 10 and 15, Clade F [11], [12] [13]. The high genetic diversity of *Toxoplasma* strains in the tropical zone of the Americas may partly explain why congenital toxoplasmosis is more symptomatic in South America than Europe, as was demonstrated in cohorts of congenitally infected children from different continents [8], [14], [15]. A comparative prospective cohort study of congenitally infected children in Brazil and Europe found that Brazilian children displayed eye lesions that were larger, more numerous, and more likely to affect the central part of the retina responsible for acute vision [7]. Anecdotal clinical cases were also reported in the literature, such as a severe atypical bilateral retinochoroiditis in a Brazilian patient, caused by a highly divergent, non-archetypal *T. gondii* strain [16].

Given the markedly different population structure of *T. gondii* in Europe and South America, it is relevant to study the implications of this diversity on human pathogenesis [17]. Therefore, we conducted a multicenter case series study in order to compare the different clinical and immunological characteristics between Colombian and French patients, collecting the same data and performing the same laboratory assays in patients with biologically confirmed OT. The findings were related to *Toxoplasma* strain genotyping and peptide-based strain serotyping in our patients.

## Materials and Methods

### Study population

We collected data from consecutive patients who consulted at the Departments of Ophthalmology at Strasbourg University Hospital (France) and Quindío University Health Center (Armenia, Colombia) between August 2008 and August 2010. Both departments were tertiary-level centers able to perform anterior chamber paracentesis. For both patient populations, a complete ocular examination was conducted, including best-corrected Snellen visual acuity, slit-lamp biomicroscopy, tonometry, and indirect ophthalmoscopy. The clinical diagnosis of OT was based on criteria previously described by G. Holland [6], [18]. Screened patients with clinically suspected OT and seropositive for anti-*Toxoplasma* immunoglobulin G (IgG) antibodies were subsequently submitted to biological investigations to assess the local presence of *Toxoplasma* DNA and/or the intraocular antibody synthesis [19] to confirm OT.

### Ethics statement

Ethics Committee/Institutional Review Board (IRB) approval were obtained from Hôpitaux Universitaires de Strasbourg (PHRC 2007/3964) and Quindio University (ACT 14, 2008/23-06). Written informed consent was obtained from all subjects.

### Clinical evaluation criteria

We analyzed the clinical characteristics of 19 French and 23 Colombian patients with active uveitis and biologically confirmed OT. Patients who were immunocompromised, suffered from other ocular infections, or received local or systemic anti-*Toxoplasma* treatment for active uveitis, were excluded. An assessment of the inflammation level and anatomic classification of uveitis was carried out according to the criteria proposed by the International Uveitis Study Group (IUSG) [20]. The size of the retinochoroidal lesions was measured in disc-diameters (dd).

### Sample collection and biological OT diagnosis

Paired samples of aqueous humor and serum were obtained from each subject at the time of clinical diagnosis for laboratory analysis. The Colombian samples were stored locally at −80°C and then shipped together on dry ice to Strasbourg for laboratory analysis. Aqueous humor samples (100–150 µL) were collected through anterior chamber paracentesis and stored, along with serum samples, at −80°C until analysis. The diagnosis of OT was first confirmed by real-time PCR detection of *Toxoplasma* DNA [21]. Positive PCR results were quantified using a standard curve with serial 10-fold dilutions from a calibrated suspension of *T. gondii* RH-Strain DNA. For PCR negative patients, immunoblot (IB) was performed in order to detect intraocular synthesis of *Toxoplasma*-specific antibodies (LDBIO Diagnosis, Lyon, France). If both PCR and IB were unconclusive, a modified Goldmann-Witmer test was used to prove intraocular specificantibody synthesis [22].

### Cytokine-Chemokine Profile measurement in aqueous humor

The Bio-Plex Human 27-Plex Cytokine Panel assay (Bio-Rad, Marne-la-Coquette, France) was used according to the manufacurer's recommendations to measure cytokine and chemokine levels in aqueous humor. The assay plate layout consisted in a standard series in duplicate (1 to 32 000 pg/mL), four blank wells and 20 µL duplicates of AqH samples, diluted to 50 µL with BioPlex Human serum diluent [23]. A set of *Toxoplasma* seropositive cataract patients were used as control, 9 Colombian and 10 French. Data were analyzed with Bio-Plex Manager TM software V1.1.

### *Toxoplasma* strain genotyping analysis

DNA extraction for genotyping analysis was performed directly on ocular fluid samples and indirectly on infected cell cultures for six reference strains. GT1, PTG, and CTG strains were selected as reference Types I, II, and III strains, respectively. TgCtCo02, TgCtCo05, and TgCtCo07 strains were selected as reference Colombian strains [24], [25]. *T. gondii* DNA samples were subjected to genotyping analysis with 15 microsatellite markers in a multiplex PCR assay, as described elsewhere [26].

### *Toxoplasma* strain serotyping analysis

Serotyping of Toxoplasma infections was performed using 5 polymorphic synthetic peptides derived from the *T. gondii* dense granule proteins (GRA), GRA6 and GRA7. This test detects the presence of strain specific antibodies raised against Type II or non-Type II GRA6/7 alleles in patients infected with Type II or non Type II (NE-II) parasites respectively, as previously described [14], [27]. Briefly, the ELISA results presented are an optical density (OD) index obtained by dividing the OD value at 405 nm for each of the 5 serotyping peptides by the mean of the OD readings for the 2 control peptides. Threshold values are determined by averaging the normalized OD ratio from 100 seronegative French samples and adding 2 standard deviations, above which normalized values are considered positive. Obtained results are divided in four populations depending on their reactivity to the 5 peptides: I/III, ATYP, no reactivity (NR), and II [28]. I/III, ATYP and NR are considered as NE-II [14]. Sera from pregnant women, tested *Toxoplasma* seropositive in our laboratories, were used to assess the *Toxoplasma* serotype in a larger population from each country, 45 serum samples from Colombia and 100 from France.

### Statistical analysis

Mann-Whitney test followed by Dunn's Multiple Comparison test was applied for comparison of clinical and laboratory characteristics for French and Colombian patients with confirmed active ocular toxoplasmosis (P values<0.05 were considered statistically significant; Stata software, College Station (Tx) USA). Fisher's exact test was used to compare diagnostic performances of IB and PCR as well as the serotype prevalence. Wilcoxon matched-pairs signed rank test was performed to compare IB patterns. Mann-Whitney test was used to compare intraocular parasite loads (P values<0.05 were considered statistically significant. Kruskal-Wallis test followed by Dunn's Multiple Comparison test were applied for comparison of cytokine and chemokine levels in aqueous humor between control and OT populations in both countries (P values<0.05 were considered statistically significant) (GraphPad Prism, La Jolla, CA, USA).

## Results

### Clinical characteristics

The clinical findings for OT patients are summarized in Tables 1 and S1. Statistically significant differences between groups were found for eight parameters, being higher in Colombian patients in all cases: i) time between consultation and anterior chamber paracentesis (p = 0.02); ii) size of active lesions (p = 0.04); iii) unilateral macular involvement (p = 0.001); iv) unilateral visual impairment (p = 0.04); v) vitreous inflammation (p = 0.00001); vi) percentage of patients with synechiae (p = 0.04); vii) vasculitis (p = 0.04) and viii) bilateral involvement (p = 0.04). In addition, there was a trend towards higher values for the Colombian patients regarding the number of lesions, number of recurrences, and intraocular pressure (IOP), although these differences were not statistically significant. We conducted a stratified analysis in order to exclude the influence of time before anterior chamber paracentesis as a possible cause of the differences in clinical findings. We compared early (<20 days after symptom onset) and late consultations (>20 days after symptom onset). As shown in Table 2 and supplementary figure 1, most significant clinical differences between the populations were also visible when comparing only the early-consultant groups.

**Table 1:** Comparative clinical and laboratory characteristics for French and Colombian patients with confirmed active ocular toxoplasmosis (all cases).

CLINICAL CHARACTERISTICS	FRANCE (n = 19)			COLOMBIA (n = 23)			P-value
	Mean/n(%)*	Median	(Range)	Mean/n(%)*	Median	(Range)	
**Age at consultation**	45.22	44.5	(16–77)	38.3	37	(20–86)	0.23
**Evolution time (days)**	15	6	(1–150)	46	15	(4–240)	**0.02**
**Macular involvement**	2 (10.53%)	N.A.	N.A.	13 (56.52%)	N.A.	N.A.	**0.001**
**Vitreous inflammation Level(+)** **	0.95	2	(0–1)	2.41	2	(0–4)	**0.00001**
**Synechia**	2 (5.26%)	N.A.	N.A.	11 (47.8%)	N.A.	N.A.	**0.04**

Mann and Whitney test followed by Bonferroni-Dunn's Multiple Comparison test was applied (P values<0.05 were considered statistically significant)

* Percentages take into account only the patients with available information

** Measured according to Standardization Uveitis Nomenclature (SUN)

N.A. = Not applicable (for categorical variables)

**Table 2:** Comparative clinical and laboratory characteristics for French and Colombian patients with confirmed active ocular toxoplasmosis, stratified by evolution time before consultation.

CLINICAL CHARACTERISTICS	EARLY CONSULTATION						
	FRANCE (n = 15)			COLOMBIA (n = 12)			P-value
	Mean/n(%)*	Median	(Range)	Mean/n(%)*	Median	(Range)	
**Age at consultation**	44.64	44.5	(16–74)	31.33	24	(20–82)	**0.05**
**Macular involvement**	2 (13.33%)	N.A.	N.A.	7(58.33%)	N.A.	N.A.	**0.01**
**Vitreous inflammation Level(+)**	0.93	2	(0–1)	2.58	2	(0–4)	**0**
**Strabismus**	0 (0%)	N.A.	N.A.	3 (25%)	N.A.	N.A.	**0.04**
**Synechia**	0.13	N.A.	N.A.	0.5	N.A.	N.A.	**0.03**

	LATE CONSULTATION						
	FRANCE (n = 4)			COLOMBIA (n = 11)			P-value
	Mean/n(%)*	Median	(Range)	Mean/n(%)*	Median	(Range)	
**Age at consultation**	47.25	45.5	(821–77)	45.9	43	(27–86)	0.9
**Macular involvement**	0(0%)	N.A.	N.A.	6(54.54%)	N.A.	N.A.	0.06
**Vitreous inflammation Level(+)**	1	2	(1–1)	2.23	2	(0–4)	0.11
**Strabismus**	0(0%)	N.A.	N.A.	0(0%)	N.A.	N.A.	0.39
**Synechia**	0	N.A.	N.A.	0.45	N.A.	N.A.	0.11

Mann and Whitney test followed by Bonferroni-Dunn's Multiple Comparison test was applied (P values<0.05 were considered statistically significant)

* Percentages take into account only the patients with available information

N.A. = Not applicable (for categorical variables)

### Detection of *Toxoplasma* DNA in aqueous humor and strain genotyping analysis

**Figure 1:**
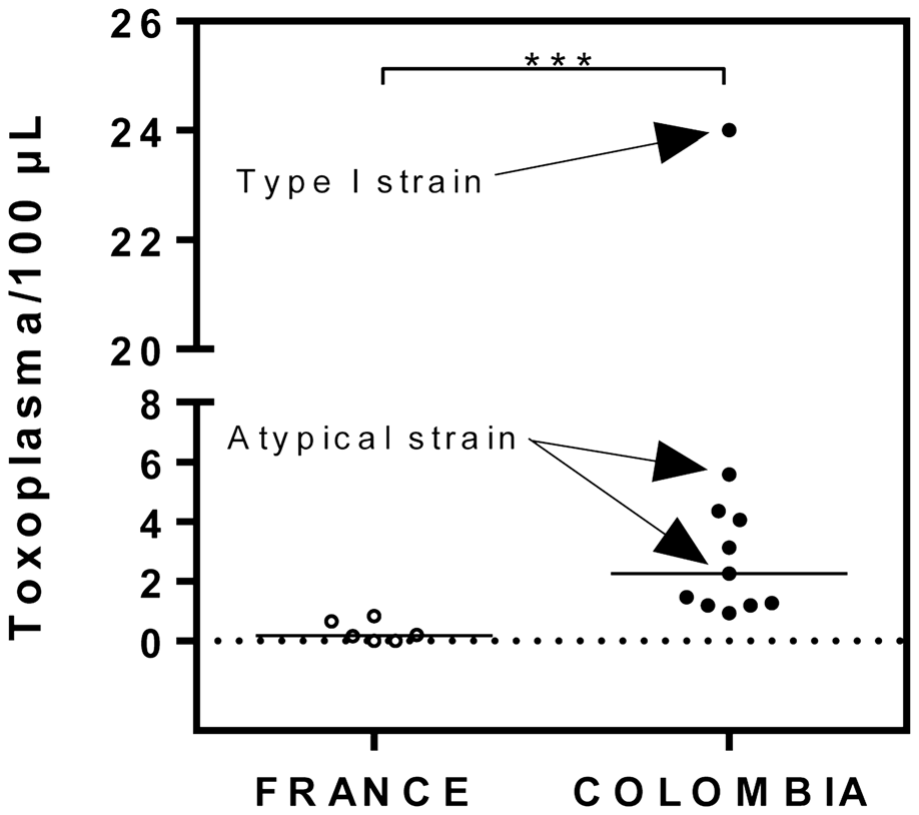
Parasite load in PCR positive patients. Aqueous humor was obtained from French and Colombian OT patients, DNA extracted, and the number of parasites per mL aqueous humor determined by quantitative PCR using *Toxoplasma*-specific primers. The Mann and Whitney test was significant (P = 0.0002).

In Colombians, aqueous humor samples revealed the presence of *T. gondii* DNA in 11 out of 23 samples (47.8%). In French patients, *T. gondii* DNA could be detected in aqueous humor samples of 7 out of 19 patients (36.8%). This difference was not statistically significant. In contrast, parasite loads in aqueous humor were significantly higher in Colombian patients, 4.53 parasites ± 2 per 100 µL versus 0.35±0.13 parasites per 100 µL (p = 0.0006) (Figure 1). Aqueous humor samples from all French patients and 14 Colombian patients had an insufficient amount of *T. gondii* DNA for genotyping analysis. Only 9 Colombian ocular fluid samples were submitted for multilocus PCR-DNA sequence genotyping analysis. Six had unsuccessful PCR amplification for all 15 tested markers due to low *T. gondii* DNA concentration. The genotype of one clinical sample (case COL-#6) was closely related to a Type I strain, but harboring unique alleles at three MS loci, M102, N83 and AA, using 15 amplified markers (Table 3). Of note, the genotype of a reference Colombian isolate (TgCtCo07) collected from a cat in 2005 was also Type I-like, suggesting that Type I-like strains may not be uncommon in animals and humans in Colombia. The genotypes of the other two clinical samples (cases COL-#26 and COL-#38) could not be fully determined, with only four and five successfully amplified markers, respectively. However, the results of the amplified markers showed that both genotypes were different from the Type II or III strains, which are common in North America and Europe. They present a majority of Type I alleles (case COL-#26), like TgCtCo07 but distinct at the N61 marker, and a combination of Type I, III, and atypical alleles (case COL-#38), like TgCtCo02 and TgCtCo05, but again distinct at the N60 and N82 genetic markers.

**Table 3:** Genotyping results of *T. gondii* DNA from 6 reference strains and 9 Colombian human ocular fluid samples with 15 microsatellite markers in a single multiplex PCR assay.

Type^a^	Isolate^b^	Origin (Host)^c^	Microsatellite markers^d^														
			*TUB2*	*W35*	*TgM-A*	*B18*	*B17*	*M33*	*IV.1*	*XI.1*	*M48*	*M102*	*N60*	*N82*	*AA*	*N61*	*N83*
I	GT1 (Reference)	USA (Goat)	291	248	209	160	342	169	274	358	209	168	145	119	265	87	306
II	PTG (Reference)	USA (Sheep)	289	242	207	158	336	169	274	356	215	174	142	111	265	91	310
III	CTG (Reference)	USA (Cat)	289	242	205	160	336	165	278	356	215	190	147	111	269	89	312
Atypical	TgCtCo02 (Reference)	Colombia (Cat)	291	248	205	160	342	167	274	358	209	166	142	123	291	89	306
Atypical	TgCtCo05 (Reference)	Colombia (Cat)	291	242	205	160	336	165	276	356	223	166	142	121	279	87	304
I	TgCtCo07 (Reference)	Colombia (Cat)	291	248	209	160	342	169	274	358	209	166	147	127	265	87	306
ND	COL-# 15)	Colombia (Human, AH)	NA	NA	NA	NA	NA	NA	NA	NA	NA	NA	NA	NA	NA	NA	NA
ND	COL-#2)	Colombia (Human, AH)	NA	NA	NA	NA	NA	NA	NA	NA	NA	NA	NA	NA	NA	NA	NA
I	COL-#6)	Colombia (Human, AH)	291	248	209	160	342	169	274	358	209	166	145	117	269	87	306
ND	COL-# 1)	Colombia (Human, AH)	NA	NA	NA	NA	NA	NA	NA	NA	NA	NA	NA	NA	NA	NA	NA
ND	COL-#24	Colombia (Human, AH)	NA	NA	NA	NA	NA	NA	NA	NA	NA	NA	NA	NA	NA	NA	NA
ND	COL-#25	Colombia (Human, AH)	NA	NA	NA	NA	NA	NA	NA	NA	NA	NA	NA	NA	NA	NA	NA
ND	COL-#26	Colombia (Human, AH)	NA	NA	209	160	NA	NA	NA	NA	NA	NA	NA	127	NA	89	NA
ND	COL-#38	Colombia (Human, AH)	NA	242	205	NA	342	NA	NA	NA	NA	NA	140	117	NA	NA	NA
ND	COL-#41	Colombia (Human, AH)	NA	NA	NA	NA	NA	NA	NA	NA	NA	NA	NA	NA	NA	NA	NA

a ND, Not Determined.

b PTG is a clone of the ME49 strain; CTG is also known as CEP or C strain. GT1, PTG, and CTG are reference type I, II, and III strains, respectively. TgCtCo02, TgCtCo05, and TgCtCo07 are reference strains isolated from cats in Colombia. All DNA samples from reference strains were kindly provided by Chunlei Su and Jitender Dubey.

c AH, Aqueous Humor; VH, Vitreous Humor.

d NA, Not Amplified

### Detection of intraocular anti-*Toxoplasma* antibodies

**Figure 2:**
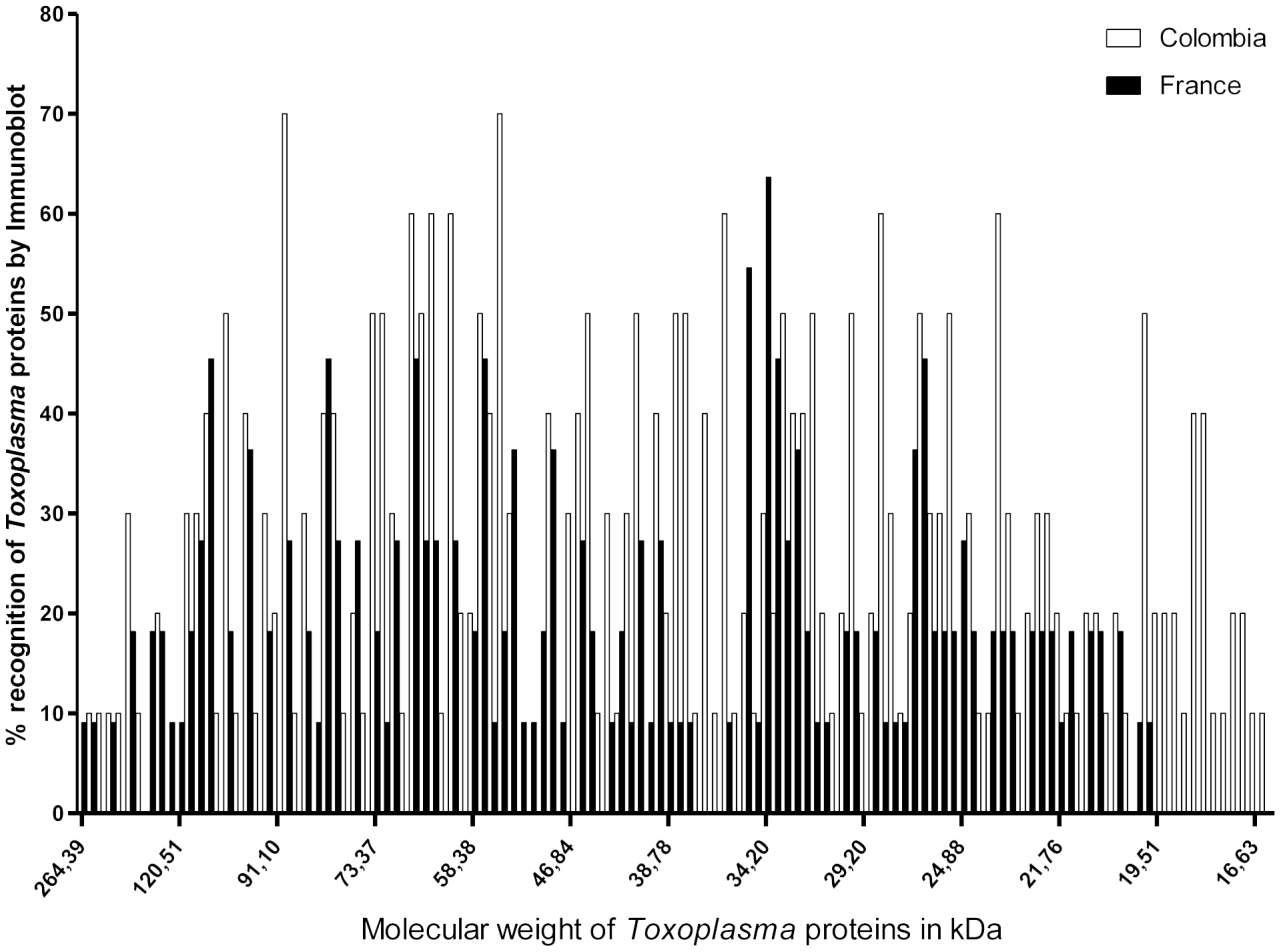
Differences in pattern recognition by immunoblotting between Colombian and French patients. Aqueous humor samples were tested against *Toxoplasma* proteins, as detailed in the *Materials and methods* section. The percentage of French or Colombian patients recognizing the different Toxoplasma proteins is given. A Wilcoxon matched-pairs signed rank test was performed to compare IB patterns (p<0.0001).

IB detected local antibody production in 19/23 Colombian (82.6%) and 13/19 French (68.4%) patients (not significant). However, a significant difference was observed in number of bands and their recognition pattern of *Toxoplasma* proteins (p<0.0001) (Figure 2). Specific proteins were recognized in 3.3% to 63.3% of Colombian patients and 3.8% to 53.8% of French patients. Colombian patients recognized most frequently a 62 kDa protein, observed in 63.3% of patients. In French patients, the most frequently detected protein was at 34.2 kDa, found in 53.8% of patients.

### *Toxoplasma* strain serotyping analysis

As the amount of aqueous humor was insufficient for *Toxoplasma* strain typing using an ELISA peptide-based assay, we decided to serotype these patients using their sera. Ten OT patients from each center were assessed, all from the early consultation group. Among the Colombian patients, no Type II serotype was detected. We found 4 I/III, one atypical and 5 non reactive (NR) serotypes (Table 4). In contrast, all tested French OT patients showed Type II serotypes except one patient with an atypical serotype. These patterns were significantly different between the two groups (p<0.0001). The two cases COL#26 and COL#38, found as suspected Type I and Type I/III by genotyping, were serotyped as NR and type I/III, respectively (Table 4).

**Table 4:** Distribution of Toxoplasma serotypes among Colombian and French OT patients (OT-CO# and OT-FR#) were assessed for antibodies reacting to 5 strain-specific GRA6 and GRA7 polymorphic peptides derived from Type II or Type I/III parasites.

PATIENTS								
Colombia								
	6I/III***	D6I/III****	6II	D6II	7II	SAG1*****	Serotype	Conclusion
OT-CO1	1.6←	1.2	1.3	1.2	1.0	7.3←	I/III	NE-II
OT-CO2	7.6←	1.3	4.1←	1.3	0.9	25.2←	ATYP	NE-II
OT-CO3*	0.1	0.8	1.1	1.0	0.9	8.3←	NR	NE-II
OT-CO4	1.6←	1.1	1.1	1.4	1.1	2.7←	I/III	NE-II
OT-CO5**	1.7←	1.5←	1.3	1.1	1.0	25.0←	I/III	NE-II
OT-CO6	1.0	0.9	1.1	1.0	0.6	4.6←	NR	NE-II
OT-CO7	5.5←	1.0	1.0	1.0	1.0	8.5←	I/III	NE-II
OT-CO8	1.3	1.0	1.1	0.6	1.0	26.6←	NR	NE-II
OT-CO9	0.8	0.8	0.7	1.0	0.6	6.0←	NR	NE-II
OT-CO10	1.2	1.3	1.2	1.0	1.0	20.9←	NR	NE-II

France								
OT-FR1	1.4	1.3	3.3←	2.0←	11.6←	26.0←	II	II
OT-FR2	1.2	1.1	2.0←	2.1←	1.5←	25.4←	II	II
OT-FR3	0.6	0.6	0.8	0.8	4.2	13.7←	II	II
OT-FR4	1.1	1.2	2.5←	3.0←	4.4←	21.4←	II	II
OT-FR5	0.0	1.2	1.6←	1.2	10.7←	4.5←	II	II
OT-FR6	1.0	1.0	1.5←	1.6←	2.3←	10.5←	II	II
OT-FR7	1.1	1.2	1.4	1.6←	1.3	6.4←	II	II
OT-FR8	1.1	1.4	3.2←	3.2←	2.4←	17.6←	II	II
OT-FR9	ND	ND	ND	ND	ND	ND	ND	ND
OT-FR10	2.4←	1.1	1.1	1.1	3.1←	8.1←	ATYP	NE-II

Peptide names were abbreviated as follows: “6” denoting peptides from GRA6; “7” from GRA7; “I/III” or “II” for the strain bearing the peptide allele; and “d” indicating a truncated diagnostic peptide. Reactivity at SAG1 served as a positive control to indicate the presence of anti-Toxoplasma antibodies. Type I/III infections produce antibodies that react with 1 or both 6-I/III and d6I/III peptides, Type II infections react with at least 1 of the 6-II, d6-II and 7-II peptides, Atypical (ATYP) infections identify strain-specific antibodies that react with both I/III and II peptides, or do not react (nonreactive “NR”) with any of the allele-specific peptides. For the purposes of statistical analyses, patients were classified as possessing either a Type II serotype or NE-II serotype (for all other reactivities). Fischer's exact test was applied for comparison between population and difference was highly significant (P<0.0001)

* found with a majority of Type I alleles by genotyping; case COL#26

** found with a combination of Type I, III, and atypical alleles by genotyping : case COL#38

*** 6I/III refers to the C-terminal peptide from the Dense Granule protein GRA6 (peptide “CLHPERVNVFDY”)

**** D″ stands for a delimited version of the 6I/III peptide, by truncation of the terminal Y amino acid, used to confirm specificity

***** SAG1 is a recombinant protein used to confirm seropositivity among the patient samples received for serotyping

← Positive reactivity by ELISA-based assay (cut-off value = 1.4)

To test if certain *T. gondii* strains are associated with OT, we determined the overall distribution of serotypes in infected non-OT control populations from both countries. Among the 45 Colombian control patients, only 6 subjects (13.3%) had a type II whereas 39 (86.6%) had NE-II serotypes, which were subdivided in 6 NR, 29 type I/III and 4 atypical serotypes. Of 100 French control patients, we found 64 (64%) type II, and 36 (36%) with NE-II; 10 NR, 2 type I/III and 24 atypical serotypes. No statistically significant differences were observed between the control and OT groups in Colombian patients, however we found a significant difference (P = 0.02) between the French control and OT populations, with respect to the proportion of the two types, II and NE-II.

### Ocular cytokine and chemokine pattern

**Figure 3:**
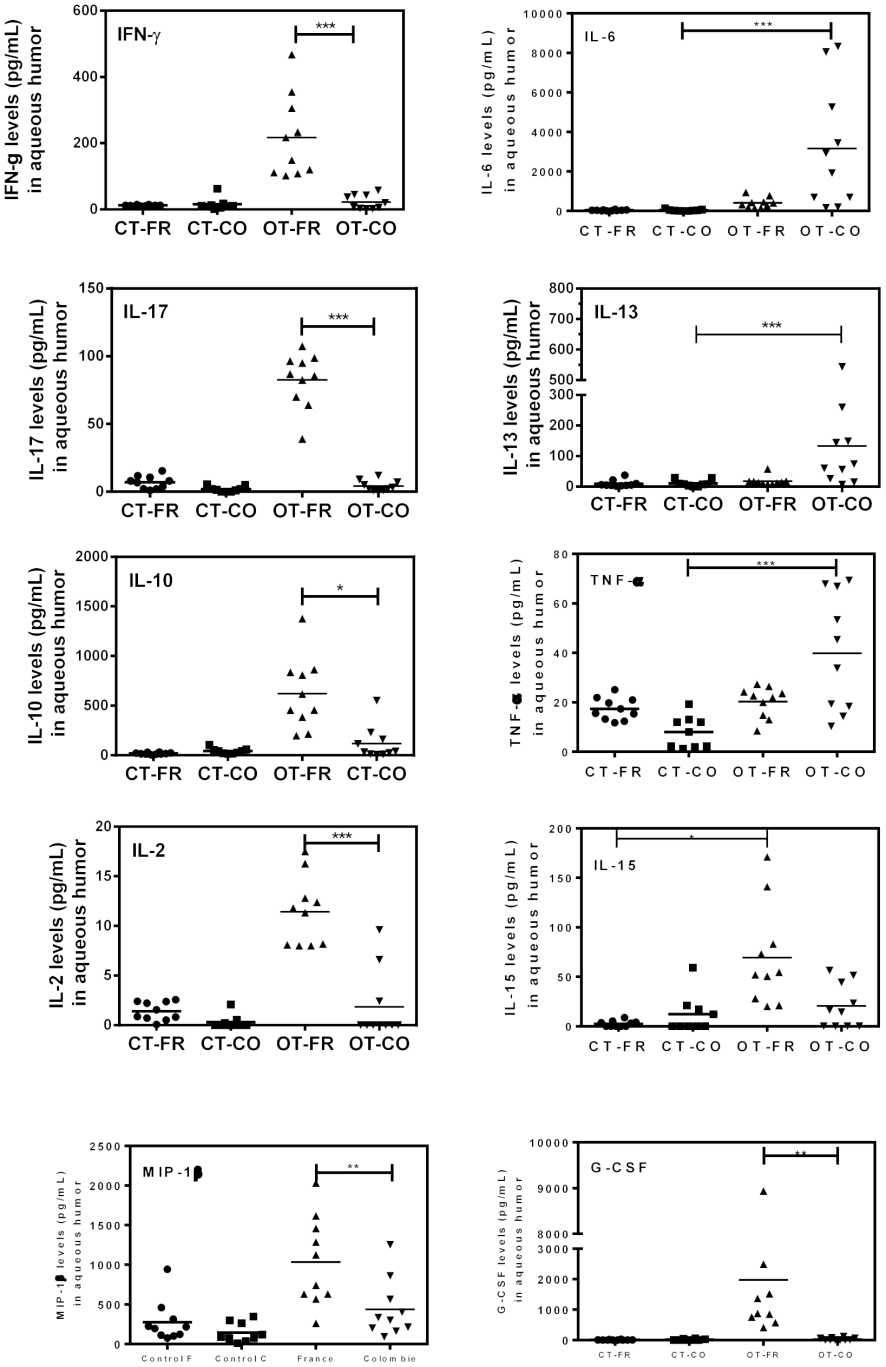
Cytokine and chemokine levels (pg/mL) in aqueous humor for French and Colombian patients. Aqueous humor samples were tested for ocular cytokines and chemokines as detailed in *Material and methods* section, for Colombian (OT-CO; n = 10) and French ocular toxoplasmosis patients (OT-FR; n = 10). They were compared to cataract control groups from Colombia (CT-CO; n = 9) and France (CT-FR; n = 10). Kruskal-Wallis test followed by Dunn's Multiple Comparison test were applied for comparison between populations (significant for P<0.05).

Cytokines patterns in aqueous humor of OT patients were compared to cataract controls (Figure 3 and Table S2 in Text S1). Several immune mediators were augmented in French, as well as in Colombian patients. In French patients, the Th1 type cytokines IFN-γ, IL-2 and IL-15 were expressed in all patients. This Th1 immune response was associated to a Th17 response with increased IL-17 production. Additionally, we observed a large proinflammatory response with increased levels of IL-6, IL-1β, IL-8, MIP-1β, MCP-1 and G-CSF. These patients also possessed a corresponding anti-inflammatory response was based on the presence of IL-4, IL-10, and IL-1RA. In contrast, Colombian patients had lower expression of major proinflammatory immune modulators, including IFN-γ, IL-15, IL-17, IL-2, IL-10, MIP-1β, GM-CSF and G-CSF, with the exception of elevated TNF-α and IL-6 levels. These patients also had elevated levels of the counterregulating Th2-type cytokine IL-13.

## Discussion

Previously published studies found differences between South American and European clinical case series on adult patients in terms of frequency of serological markers in OT [8], probability of symptoms in congenital infection [7], as well as inflammation levels and IOP [9]. However, these were mostly retrospective evaluations of multiple studies. Their main limitation is their inclusion of patients with “suspected” OT, rather than biologically confirmed cases. While the ocular signs of toxoplasmic retinochoroiditis are highly suggestive of this disease, they may be mimicked by other infections [22], while in some cases, the symptoms may be atypical [19], [29]. Therefore, we strengthened our evaluation by inclusion of biologically confirmed OT cases only, as well as by comparing the same bio-clinical data from two different populations of OT patients, located in South America and Europe in a cross sectional study. Among the 17 criteria analyzed in the two populations, the following were significantly higher in Colombian patients: macular involvement, vitreous inflammation, strabismus, bilateral involvement and synechiae. Our findings confirm and expand the data from the retrospective study of Dodds *et al.* from patients with biologically unconfirmed OT which found elevated IOP, increased presence of synechiae, AC cells, flare, and vitreous humor haze [9]. In our study, one key difference between the two patient populations was the date of consultation, as Colombian patients consulted later than the French. However, when our analysis was stratified regarding this aspect, the observed clinical differences remained significant.

The main hypothesis for these clinical differences is based on the idea that severe disease in humans may result from poor host adaptation to neotropical zoonotic strains of *T. gondii* [11]. Our study accumulated some clues supporting this hypothesis.

Central strain-specific parasite virulence factors in human infections were revealed in the last years [30]. Their role in the presence of more virulent parasite genotypes in South America [11], [12] is not yet thoroughly studied. Theses strains are rarely found in Europe [31] where Type II genotypes predominate, including in OT patients [32]. In the three Colombian OT patients where we could detect *Toxoplasma* DNA, we found one Type I and two atypical strains. The fact that no patient of the French group had a sufficient ocular parasite load for genotyping clearly shows the difference in ocular virulence. Additionally, we noticed that intraocular antibodies responses showed major differences in *Toxoplasma* antigen recognition by an immunoblotting assay. Even if this could be partly due to better detection of Toxoplasma Type I antigens used in this assay by Colombian patients, other, host immune specific factors are certainly crucial such as local antibodies, whose exact role and function should be explored.

Our serotyping assay confirmed that Colombian and French patients recognize different strain-specific epitopes. Colombian OT patients recognized a heterogeneous pattern of strain specific peptides, but none of them were from type II strains. The French OT patients recognized only Type II strain specific peptides, confirming the reliability of this test in a geographic region with predominant type II strains infections [33]. The corresponding control populations presented the same serological pattern for Colombia, but a slightly different pattern for France, where some sera were non reactive to Type II antigens. The difference may due to the unequal sample sizes, so this point needs further investigation using more samples and equilibrated OT and control population. However, these data indicate that type II and non-type II strains are able to cause ocular pathology, but with a markedly different clinical picture. Concerning the Colombian strains, current serotyping techniques might be not sensitive enough to distinguish the highly variable strains.

When we looked at the patients' local immunological reaction, we observed clearly different cytokine signatures. In French patients, the host-parasite relationship seems to be equilibrated between protection and inflammation. The protective effect of IFN-γ is balanced by anti-inflammatory cytokines such as IL-2 and IL-10. The role of IL-17 is controversial. We have previously observed an early pathologic and parasite promoting role for IL-17 in French patients and in an animal model infected by a Type II *Toxoplasma* strain [34]. In the intraocular ocular environment, IL-17 would attract neutrophils [35] and, accompanied by IL-15 and MIP-1β/CCL4, activates and attracts NK cells [36] and monocytes [37]. All these innate immune cells might cause retinal inflammation, but then permit to control *Toxoplasma* proliferation [38], [39]. As our recent findings implicate IL-27 and the Treg subset in counterbalancing deleterious inflammatory Th17 type responses [34], the corresponding mediators deserve to be examined more closely in future studies.

In contrast, in the clinically more severe Colombian cases, IFN-γ and other major immunomodulators such as IL-17 were barely detectable, while IL-6 and IL-13 were enhanced. Virulent strains encode virulence factors able to modulate multiple immune host cell signaling pathways through polymorphic effectors secreted into the host cells such as ROP16 and GRA15 [38], [40]. The presence of *Toxoplasma* effector proteins from virulent strains could explain the down-regulation of ocular IFN-γ, leading to higher ocular parasite loads in Colombian patients. The IL-17 down-regulation remains to be explained, but decreased levels of IL-17 and other immune modulators, including proangiogenic factors, could lead to a defect in the migration of leukocytes to the eyes and be another explanation for impaired control of parasites in the context of virulent South American infections. IL-6 could also antagonize the anti-microbial properties of IFN-γ by sustained activation of STAT3, a potent inhibitor of IL-12 and IFN-γ [41]. Down-regulation of IFN-γ and its anti-*Toxoplasma* activity was also observed for IL-13 in human fibroblasts [42]. It is important to note here that Type I strains express a ROP16 allele associated with prolonged activation of STAT3 and STAT6 signaling, which may in part contribute to the increased IL-13 levels, whereas Type II strains activate this pathway only transiently, allowing the establishment of an inflammatory reaction [43]. This may constitute the fundamental basis for the differential cytokine response observed in our study.

The theory of local T cell exhaustion may be also of interest in the settings of Colombian patients. Immune exhaustion is characterized by the modification of the CD8+ functions by reducing their polyfunctionality and their efficacy [44]. Indeed, high *Toxoplasma* loads associated with a decreased IFN-γ and IL-15 production and enhancement of TNF-α could be one aspect of this loss of CD8+ T cell polyfunctionality. In contrast, in French patients, elevated IL-15 is critical for homeostasis of memory CD8 T cells, and may lead to a better control of parasite proliferation and subsequent parasite latency in the retina.

Taken together, our results indicate that virulent strains observed in South America may suppress host-protective pathways, opening the way to multiplication and cytolytic activity of the parasite in retinal tissues including blood vessels. The presence of TNF-α in most of these patients could also contribute by enhancing an ongoing immunopathological retinal process [45]. In contrast, in French patients, the cytokinic environment may lead to the encystation of the parasite in the retinal tissues, leading to subsequent recurrences.

Of course, for ethical reasons, we were only able to take one time-point. Our results represent thus a snapshot of a developing immune response. Additionally, a multifactorial origin of the observed clinical and biological differences could not be excluded. In our study, the source of contamination may have been drinking water collected from surface water sources (*i.e.*, rivers, lakes) [46], [47], [48], [49]. The more common macular involvement in Colombian patients is often associated with congenital toxoplasmosis [6], [15], [50], [51]. Even if we studied adult populations, we cannot exclude a congenital origin of infection in some Colombian patients. Moreover, acute toxoplasmosis was only diagnosed in 2 Colombian and 1 French case. The remaining population was considered to exhibit chronic toxoplasmosis. Finally, individual susceptibility was previously related to variations in various genes encoding immune response players, such as IFN-γ, IL-1α, IL-10, TLR-9 or ABCA4, COL2A1, and P2X_7_-R [52], [53], [54], [55]. These genetically susceptible patients are possibly less able to cope with a more virulent strain. Further investigations with larger cohorts including an evaluation of their immunological response and their individual susceptibility to *Toxoplasma* are needed to address these topics.

## Supporting Information

Text S1 **Checklist S1.** Strobe checklist for a cross sectional study, including 19 French and 23 Colombian cases of confirmed active ocular toxoplasmosis. Clinical, parasitological and immunological responses are compared and correlated to the infecting strains. **Figure S1.** Fundus examination in a patient with bilateral-extensive-multiple, central and peripheral, chorio-retinal scars (white circled lesions) in a Colombian patient suffering from a severe ocular toxoplasmosis; A : right eye; B : left eye. **Table S1.** Complete data of all clinical and laboratory characteristics. Mann and Whitney test followed by Bonferroni-Dunn's Multiple Comparison test was applied (P values<0.05 were considered statistically significant). **Table S2.** Intraocular cytokines, chemokines and growth factors in aqueous humor of Cataract Control patients from France (CT-CO) and Colombia (CT-FR) and from Ocular toxoplasmosis patients from France (OT-FR) and Colombia (OT-CO). Levels of these immune mediators are expressed as mean and standard deviation, median and range (min-max) in pg/mL. Statistical differences between CT and OT and between OT from France versus OT from Colombia were calculated using a Kruskal-Wallis test followed by Dunn's Multiple Comparison test. Significant differences between populations (P<0.05) were highlighted by tinting the spaces. Description of major general functions of cytokines and chemokines are issued from “Commins SP et al., J Allerg Clin Immunol, 2010; Banchereau J. et al., Nature Immunology, 2012”.
(DOCX)
