# Supplementary material for: Severe South American Ocular Toxoplasmosis Is Associated with Decreased Ifn-γ/Il-17a and Increased Il-6/Il-13 Intraocular Levels
Source: PLoS Negl Trop Dis. 2013 Nov 21;7(11):e2541. doi: 10.1371/journal.pntd.0002541 (PMC3837637; doi:10.1371/journal.pntd.0002541)
Supplement: Text S1 — Checklist S1. Strobe checklist for a cross sectional study, including 19 French and 23 Colombian cases of confirmed active ocular toxoplasmosis. Clinical, parasitological and immunological responses are compared and correlated to the infecting strains. Figure S1. Fundus examination in a patient with bilateral-extensive-multiple, central and peripheral, chorio-retinal scars (white circled lesions) in a Colombian patient suffering from a severe ocular toxoplasmosis; A : right eye; B : left eye. Table S1. Complete data of all clinical and laboratory characteristics. Mann and Whitney test followed by Bonferroni-Dunn's Multiple Comparison test was applied (P values<0.05 were considered statistically significant). Table S2. Intraocular cytokines, chemokines and growth factors in aqueous humor of Cataract Control patients from France (CT-CO) and Colombia (CT-FR) and from Ocular toxoplasmosis patients from France (OT-FR) and Colombia (OT-CO). Levels of these immune mediators are expressed as mean and standard deviation, median and range (min-max) in pg/mL. Statistical differences between CT and OT and between OT from France versus OT from Colombia were calculated using a Kruskal-Wallis test followed by Dunn's Multiple Comparison test. Significant differences between populations (P<0.05) were highlighted by tinting the spaces. Description of major general functions of cytokines and chemokines are issued from “Commins SP et al., J Allerg Clin Immunol, 2010; Banchereau J. et al., Nature Immunology, 2012”. (DOCX) [file pntd.0002541.s001.docx]

**Supplementary data Table S1 in Text S1:** Complete data of all clinical and laboratory characteristics. Mann and Whitney test followed by Bonferroni-Dunn’s Multiple Comparison test was applied (P values < 0.05 were considered statistically significant).

| **CLINICAL CHARACTERISTICS** | | | **FRANCE (n = 19)** | | | **COLOMBIA (n = 23)** | | |  |
| --- | --- | --- | --- | --- | --- | --- | --- | --- | --- |
|  |  | | Mean /n (%)* | Median | (Range) | Mean /n( %)* | Median | (Range) | **P-value** |
| **AGE** | **Age at consultation** | | 45.22 | 44.5 | (16-77) | 38.3 | 37 | (20-86) | 0.23 |
|  | **Age at first episode** | | 40 | 42 | (1-70) | 33.96 | 29 | (16-85) | 0.46 |
|  | **Age at last acute episode** | | 47.29 | 46 | (23-76) | 39.45 | 37.5 | (20-86) | 0.34 |
|  |  | |  |  |  |  |  |  |  |
| **GENDER** | **Male** | | 12 (63.16%) | N.A. | N.A. | 14 (60.87%) | N.A. | N.A. | 0.95 |
|  | **Female** | | 7 (36.84%) | N.A. | N.A. | 9 (39.13%) | N.A. | N.A. | 0.21 |
|  |  | |  |  |  |  |  |  |  |
| **OCULAR INVOLVEMENT** | **Right eye** | | 8 (42.10%) | N.A. | N.A. | 14 (60.87%) | N.A. | N.A. | 0.23 |
|  | **Left eye** | | 11 (57.89%) | N.A. | N.A. | 10 (43.47%) | N.A. | N.A. | 0.36 |
|  | **Bilateral** | | 1 (5.56%) | N.A. | N.A. | 7 (30.43%) | N.A. | N.A. | **0.04** |
|  | |  |  |  |  |  |  |  |  |
| **EVOLUTION TIME** | | **(days)** | 15 | 6 | (1-150) | 46 | 15 | (4-240) | **0.02** |
|  |  | |  |  |  |  |  |  |  |
| **LOCALIZATION OF INFLAMMATION** | **Panuveitis** | | 10 (52.63%) | 1 | (0-1) | 11 (47.82%) | N.A. | N.A. | 0.76 |
|  |  | |  |  |  |  |  |  |  |
| **NUMBER OF RETINOCHOROIDAL LESIONS** | **Active lesions** | | 1 | 1 | (1-1) | 1.22 | 1 | (0-4) | 0.3 |
|  | **Inactive lesions** | | 1.05 | 1 | (0-5) | 1.48 | 1 | (0-4) | 0.36 |
|  | **Total (active and inactive)** | | 1.63 | 2 | (0-6) | 2.35 | 2 | (0-6) | 0.16 |
|  |  | |  |  |  |  |  |  |  |
| **SIZE OF LESIONS** | **Size of active lesions (dd)** | | 1.26 | 1 | (0.5-5) | 1.93 | 2 | (0-5) | **0.04** |
|  | **Size of Inactive lesions (dd)** | | 1.27 | 1 | (0.5-3) | 1.07 | 1 | (0-5) | 0.21 |
|  |  | |  |  |  |  |  |  |  |
| **LESION LOCALIZATION** | **Macular** | | 2 (10.53%) | N.A. | N.A. | 13 (56.52%) | N.A. | N.A. | **0.001** |
|  | **Posterior non-macular pole** | | 12 (63.16%) | N.A. | N.A. | 12 (52.17%) | N.A. | N.A. | 0.49 |
|  |  | |  |  |  |  |  |  |  |
| **INFLAMMATION LEVEL** | **AH inflammation (+)** | | 1.5 | 1 | (0-3) | 1.83 | 2 | (0.5-4) | 0.34 |
|  | **Vitreous inflammation (+)** | | 0.95 | 2 | (0-1) | 2.41 | 2 | (0-4) | **0.00001** |
|  | | |  |  |  |  |  |  |  |
| **NUMBER OF RECURRENCES** | | | 1.32 | 1 | (0-7) |  | 1.83 | 1 | (0-9) |
|  |  | |  |  |  |  |  |  |  |
| **SCAR NUMBER** | | | 1.11 | 1 | (0-5) |  | 1.65 | 1 | (0-4) |
|  |  | |  |  |  |  |  |  |  |
| **BCVA** | **Legal blindness in one eye (BCVA <20/200 )** | | 4 (23.53%) | N.A. | N.A. | 9 (39.13%) | N.A. | N.A. | 0.31 |
|  | **Legal blindness in one both eyes (BCVA <20/200 )** | | 0 (0%) | N.A. | N.A. | 1 (4.34%) | N.A. | N.A. | 0.38 |
|  | **OD BCVA 20/20-20/40** | | 12 (63.16%) | N.A. | N.A. | 12 (52.17%) | N.A. | N.A. | 0.48 |
|  | **OD BCVA 20/40-20/200** | | 4 (21.05%) | N.A. | N.A. | 7 (30.43%) | N.A. | N.A. | 0.5 |
|  | **OD BCVA <20/200** | | 3 (16.67%) | N.A. | N.A. | 5 (21.73%) | N.A. | N.A. | 0.69 |
|  | **OS BCVA 20/20-20/40** | | 16 (88.89%) | N.A. | N.A. | 10 (43.47%) | N.A. | N.A. | **0** |
|  | **OS BCVA 20/40-20/200** | | 1 (5.56%) | N.A. | N.A. | 7 (30.43%) | N.A. | N.A. | **0.04** |
|  | **OS BCVA <20/200** | | 1 (5.6%) | N.A. | N.A. | 5 (21.74%) | N.A. | N.A. | 0.15 |
|  |  | |  |  |  |  |  |  |  |
| **COMPLICATIONS** | **Strabismus** | | 0 (0%) | N.A. | N.A. | 3 (13.04%) | N.A. | N.A. | 0.11 |
|  | **Cataracts** | | 3 (15.78%) | N.A. | N.A. | 3 (13.04%) | N.A. | N.A. | 0.81 |
|  | **Synechiae** | | 2 (5.26%) | N.A. | N.A. | 11 (47.8%) | N.A. | N.A. | **0.04** |
|  | **Papilitis** | | 4 (21.05%) | N.A. | N.A. | 6 (26.09%) | N.A. | N.A. | 0.71 |
|  | **Vasculitis** | | 1 (5.26%) | N.A. | N.A. | 7(30.43%) | N.A. | N.A. | **0.04** |
|  | **High IOP** | | 2 (10.52%) | N.A. | N.A. | 7 (30.43%) | N.A. | N.A. | 0.12 |
|  | **CME** | | 1 (5.26%) | N.A. | N.A. | 9 (39.13%) | N.A. | N.A. | 0.35 |
|  |  | |  |  |  |  |  |  |  |
| **ACQUISITION OF INFECTION** | **Acquired infection** | | 5 (26.31%) | N.A. | N.A. | 3 (13.04%) | N.A. | N.A. | 0.29 |
|  | **Congenital** | | 1 (5.26%) | N.A. | N.A. | 1 (4.34%) | N.A. | N.A. | 0.89 |
|  | **Undetermined** | | 13 (68.42%) | N.A. | N.A. | 19 (86.96%) | N.A. | N.A. | 0.15 |
|  |  | |  |  |  |  |  |  |  |
| **TREATMENT** | **Previous treatment** | | 4 (33.33%) | N.A. | N.A. | 12 (52.17%) | N.A. | N.A. | 0.3 |
|  | **Antibiotics or antiparasitics only** | | 0 (0%) | N.A. | N.A. | 7 (30.43%) | N.A. | N.A. | N.A. |
|  | **Antibiotics or antiparasitics + Steroids** | | 4 (66.66%) | N.A. | N.A. | 7 (30.43%) | N.A. | N.A. | 0.11 |
|  | **SC steroids** | | 2 (33.33%) | N.A. | N.A. | 5 (21.73%) | N.A. | N.A. | 0.57 |
|  | **PO steroids** | | 3 (50%) | N.A. | N.A. | 9 (41.38%) | N.A. | N.A. | 0.64 |
|  | **Number of patients with additional complications** | | 1.06 | 0 | (0-10) | 2.05 | 0 | (0-8) | 0.31 |
|  |  | |  |  |  |  |  |  |  |
| **LABORATORY TESTS** | **Quantitative IgG (UI/ml)** | | 158.03 | 98 | (17.4-1178) | 170.12 | 180.9 | (36.4-301.4) | 0.83 |
|  | **Qualitative IgM** | | 19 (15.78%) | N.A. | N.A. | 23 (86.95%) | N.A. | N.A. | 0.49 |
|  | **PCR** | | 13 (39.39%) | N.A. | N.A. | 11 (47.8%) | N.A. | N.A. | 0.53 |
|  | **IB** | | 22(68.75%) | N.A. | N.A. | 19 (82.6%) | N.A. | N.A. | 0.25 |

* Percentages take into account only the patients with available information

AH: aqueous humor; BCVA: best corrected visual acuity; CME: cystoid macular edema; IB: immunoblotting; IgG: immunoglobulin G; IgM: immunoglobulin M; OD: *oculus dexter* (right eye); OS: *oculus sinister* (left eye); PCR: polymerase chain reaction

N.A.: Not applicable (for categorical variables)

**Supplementary data Table S2 in Text S1:** Intraocular cytokines, chemokines and growth factors in aqueous humor of Cataract Control patients from France (CT-CO) and Colombia (CT-FR) and from Ocular toxoplasmosis patients from France (OT-FR) and Colombia (OT-CO). Levels of these immune mediators are expressed as mean and standard deviation, median and range (min-max) in pg/mL. Statistical differences between CT and OT and between OT from France versus OT from Colombia were calculated using a Kruskal-Wallis test followed by Dunn’s Multiple Comparison test. Significant differences between populations (P<0.05) were highlighted by tinting the spaces. Description of major general functions of cytokines and chemokines are issued from “Commins SP et al., J Allerg Clin Immunol, 2010 ; Banchereau J. et al., Nature Immunology, 2012”.

|  | **CT-FR**  Mean±sd  Median  Min-max | **CT-CO**  Mean±sd  Median  Min-max | **OT-FR**  Mean±sd  Median  Min-max | **OT-CO**  Mean±sd  Median  Min-max | **CT-FR versus**  **OT-FR** | **CT-CO versus**  **OT-CO** | **OT-FR versus**  **OT-CO** | **Major general functions*** |
| --- | --- | --- | --- | --- | --- | --- | --- | --- |
| **IFN-γ** | 12.95±1.56  11.98  10.11-14.90 | 15.52±18.32  10.80  1.3-62.80 | 217±125  183  102-318.3 | 21.95±21.48  13.30  1.8-57.30 | ** | ns | *** | Th1, inhibits intracellular pathogen, such *Toxoplasma gondii*, replication |
| **IL-12 (p70)** | 0.70±0.94  0.5  0-3 | 24.07±16.98  26.65  0-53.90 | 42.36±45.53  25  7.6-142.5 | 63.62±14.18  16.15  0-38.28 | ** | ns | ns | Th1 cytokine, differentiates naive T cells into Th1 cells |
| **TNF-α** | 17.34±4.46  16.45  11.76-25.08 | 8.02±6.4  8  1.3-19.30 | 20.27±6.19  22.29  8.5-27.40 | 39.85±23.65  39.55  10.30-69.30 | ns | *** | ns | Th1/Th2 cytokine, inflammation, induce fever, apoptosis, through IL1 and IL6 production. |
| **IL-2** | 1.41±0.92  1.22  0.07-2.57 | 0.32±0.69  0  0-2.1 | 11.44±3.47  11.57  8-17.50 | 1.86±3.44  0  0-9.6 | ns | ns | *** | TH1/Th2 cytokine, growth, proliferation, and differentiation of T cells, maturation of T-regs |
| **GM-CSF** | 0.75±1.20  0  0-3.5 | 6.51±18.98  0  0-57.10 | 35.38±35.57  10  1-89 | 3.81±12.05  0  0-38.10 | * | ns | *** | Th-1/TH2 cytokine, a white blood cell growth factor |
| **IL-4** | 1.42±1.92  0.7  0-5.7 | 2.3±3.5  0.5  0-11 | 23.97±15.19  17  10.10-58-80 | 17.60±12.84  17  3.5-36.50 | *** | * | ns | TH2/Th9 cytokine, stimulates activated B-cell and T-cell proliferation, differentiates B cells into plasma Cells producing IgE; decreases the production of Th1 cells, macrophages, IFN-γ , and IL-12. |
| **IL-5** | 0.04±0.08  0  0-0.2 | 2.80±3.00  2.25  0-8 | 9.12±7.75  5.2  1.9-24.90 | 25.10±26.95  10.50  0.5-66.50 | *** | ns | ns | Th2 cytokine stimulates B cell growth and increases immunoglobulin secretion, mediates eosinophil activation |
| **IL-13** | 10.02±11.31  5.42  2.07-37-70 | 10.67±10.91  8.50  0-29 | 17.53±14.66  14  8.3-58 | 133.1±163.7  66.75  6.5-543.5 | ns | ** | ns | Th2, anti-inflammatory cytokines and induces matrix metalloproteinases (MMPs) |
| **IL-10** | 18.45± 8.98  16.50  5-32 | 43.36± 28.68  42.90  15.4-106-4 | 621.8 ±359.9  537.8  198-1377 | 117.5± 169.7  34.40  7.9-550.4 | *** | ns | * | Treg/Th3, anti-inflammatory cytokine, down-regulates Th1 and Th17 cells |
| **IL-9** | 28.58±19.24  25.90  5.8-62 | 9.4±11.24  0.45  0-20.4 | 25.34±5.85  27  18-33.5 | 7±8.06  2.7  0-16.15 | ns | ns | ** | Th9 /Th2 cytokine, stimulates cell proliferation and prevents apoptosis |
| **IL-17** | 7 ±4.7  7.4  1.11-15.48 | 1.87± 2.05  1.4  0-5.4 | 82.59±20.17  86.25  39-107.4 | 4.21±3.88  2.5  0.4-11.90 | * | ns | *** | Th17 proinflammatory cytokine |
| **IL-6** | 36.47±24.51  37.19  8.8-83.66 | 38.71±42.34  24.6  0.3-135.8 | 412.1±266  367.1  154-950 | 3163±3.105  2418  158-8330 | * | *** | ns | Inflammatory and anti-inflammatory cytokine, activates B lymphocyte, responsible of pyrexia, production of acute phase protein and apoptosis |
| **IL-15** | 2.46± 3.02  0  0-8.8 | 12.18±19.58  0  0-59.3 | 69.40±50.58  54.5  20-112 | 20.58±22.46  15.3  0-56.3 | *** | ns | ns | NK cell maturation, facilitate production of immunoglobulins and maintains survival of CD8^+^ memory T cells |
| **IL-7** | 0.42±0.54  0.2  0-1.7 | 2.67±1.75  2.65  0-5.9 | 13.72±4.11  13.3  9.4-24.5 | 8.71±8.98  6.15  0-23.9 | *** | ns | ns | B lymphocyte maturation |
| **IL-1β** | 8.48±4.88  0  0-3.5 | 13.11± 16.67  0  0-57.1 | 22.65± 23.46  10  1-89 | 26.30± 32.60  0  0-38.1 | * | ns | *** | Inflammation, involved in cell proliferation, differentiation, and apoptosis |
| **IL-1RA** | 14.02±24.77  6.8  2.5-84.3 | 4.78±5.97  4.3  0-62.3 | 73.39±41.31  70.5  35-151 | 52.52±56.79  8.3  1.3-170.3 | ** | ns | ns | Anti-inflammatory cytokine, inhibits the activities of IL-1s |
| **IL-8 /CXCL8** | 38.02±33.97  31.05  0.3-114 | 88.54±188.6  30.8  0-588.8 | 1311±720  1179  362.8-2385 | 1083±1383  562.8  42.3-4539 | *** | * | ns | Inflammation , recruits polymorphonuclear leukocytes |
| **MIP-1α/CCL3** | 10.10±6.71  7.9  2.3-21.5 | 25.38±47.41  6.1  0-145.1 | 34.8±34.53  30.5  8-111.8 | 193.2±225.2  54.6  6.1-520.6 | ns | ns | ns | Inflammation, recruits and activates polymorphonuclear leukocytes |
| **MIP-β/CCL4** | 276.1±261.4  215.5  75.5-943.5 | 145.6±123.3  98.60  0-875.6 | 1035±559  1125  568-2033 | 436±363  204.9  16.6-1250 | * | ns | ** | Inflammation, recruits monocytes/macrophages and NK cells |
| **MCP-1/CCL2** | 189±72  203  36.91-282.9 | 193±115  236.5  0-2071 | 1121±455  1170  640-1999 | 915±925  480.1  153.1-2711 | *** | ns | ns | Inflammation, recruits monocytes, memory T cells, and dendritic cells |
| **IP-10/CXCL10** | 9374±5498  10767  1461-19251 | 14901±26187  3309  0-20550 | 16543±3226  15832  12704-22477 | 10438±7575  7487  1762-20329 | ns | ns | ns | Inflammation, angiogenesis; recruits monocytes/macrophages, T cells, NK cells, and dendritic cells, |
| **G-CSF** | 7.58± 9.04  3.95  0.7-28.7 | 15.32± 21.91  0.9  0-54.48 | 1836± 2571  879  411-8939 | 32.69± 41.89  5.8  0-116.3 | ** | ns | ** | Growth Factor, survival, proliferation, differentiation of neutrophils |
| **FGF** | 33.32±22.09  32  0-68.8 | 11.17±11.11  7.75  0-30.5 | 24.91±23.90  25.5  0-49 | 21.20±17.24  20.5  0-58.5 | ns | ns | ns | Growth Factor, angiogenesis |
| **VEGF** | 701±134  699  565.8-1027 | 269±167  305.6  0-546.6 | 980±297  1008  611-1314 | 613±1020  280.6  61.6-8470 | ns | ns | ** | Growth Factor, angiogenesis |
| **PDGF** | 12.08±6.18  11.05  4.8-24.5 | 4.08±4.49  1.85  0-11.6 | 36.74±32.07  34.8  1.8-117 | 14.82±18.20  6.1  0-52.6 | ns | ns | ns | Growth factor, angiogenesis |
| **RANTES** | 127.5 ± 285.7  55.9  0-1031 | 0.2 ± 0.307  0  0-0.8 | 143.2 ± 183.4  74.5  5.5-536.3 | 570.2 ± 758.7  360.1  0-2440 | ns | * | ns |  |
| **Eotaxin** | 57.56±74.13  46.3  0-221.3 | 9.72±9.85  7.75  0-30.5 | 24.37±24.21  14.1  5.3-87 | 21.8±16.51  20.5  0-58.5 | ns | ns | ns | Eosinophil chemoattractant |

**.**
